# Immuno-Stimulatory Peptides as a Potential Adjunct Therapy against Intra-Macrophagic Pathogens

**DOI:** 10.3390/molecules22081297

**Published:** 2017-08-04

**Authors:** Tânia Silva, Maria Salomé Gomes

**Affiliations:** 1 i3S—Instituto de Investigação e Inovação em Saúde, Universidade do Porto, Porto 4200-135, Portugal; tania.silva@ibmc.up.pt; 2 IBMC—Instituto de Biologia Molecular e Celular, Universidade do Porto, Porto 4200-135, Portugal; 3 ICBAS—Instituto de Ciências Biomédicas Abel Salazar, Universidade do Porto, Porto 4050-313, Portugal

**Keywords:** antimicrobial peptide, host-defense peptide, infection, *Mycobacterium*, defensin, cathelicidin, macrophage

## Abstract

The treatment of infectious diseases is increasingly prone to failure due to the rapid spread of antibiotic-resistant pathogens. Antimicrobial peptides (AMPs) are natural components of the innate immune system of most living organisms. Their capacity to kill microbes through multiple mechanisms makes the development of bacterial resistance less likely. Additionally, AMPs have important immunomodulatory effects, which critically contribute to their role in host defense. In this paper, we review the most recent evidence for the importance of AMPs in host defense against intracellular pathogens, particularly intra-macrophagic pathogens, such as mycobacteria. Cathelicidins and defensins are reviewed in more detail, due to the abundance of studies on these molecules. The cell-intrinsic as well as the systemic immune-related effects of the different AMPs are discussed. In the face of the strong potential emerging from the reviewed studies, the prospects for future use of AMPs as part of the therapeutic armamentarium against infectious diseases are presented.

## 1. Introduction

The management of infectious diseases faces an enormous challenge due to the rampant emergence of resistant and multidrug-resistant “super-bugs”. Antibiotic resistance puts at risk even the treatment of common infections. For instance, 77 countries reported the failure of the treatment for gonorrhea using last-resort drugs (third-generation cephalosporins) and throughout the world *Klebsiella pneumoniae* fails to respond to last-resort treatments (carbapenems) [1,2]. Last year, 100 countries communicated the occurrence of extensively drug-resistant tuberculosis (XDR-TB, simultaneously resistant to isoniazid, rifampin, one fluoroquinolone and at least one of the second-line drugs) [3]. Additionally, methicillin-resistant *Staphylococcus aureus* (MRSA), responsible for a high percentage of hospital-acquired infections, are now spreading to outside hospital settings [3,4].

The treatment of infections is thus increasingly costly and prone to failure, contributing to a terrible economic burden upon public health worldwide. Aggravating the problem, antimicrobial drug development is not keeping pace with the appearance of resistant pathogens. In the last 40 years, only three new classes of antibiotics for human use were discovered, one of which limited to topical application [5,6].

Considering all this, we must acknowledge that we are moving towards a post-antibiotic era, where new antimicrobial strategies, including host-directed therapies, must emerge. In this context, antimicrobial peptides (AMPs) represent a new potential alternative to be considered for fighting infectious diseases.

## 2. Antimicrobial Peptides as Host Defense Peptides

Antimicrobial peptides (AMPs) are a large group of compounds that exhibit antimicrobial activity against several pathogens. AMPs are defined as small peptides (typically less than 50 amino acids), the majority of which have a positive charge at neutral pH (due to the presence of arginine and lysine residues), and about 50% of hydrophobic amino-acids. They are produced by almost all living organisms, as a primitive and conserved part of their immune systems [7]. AMP production can be constitutive or induced in response to inflammation, infection or injury, depending on the organism, cell type and peptide. They are produced by different blood cells, such as neutrophils, eosinophils and platelets, and also by other cell types found at sites frequently exposed to pathogens, such as the skin or mucosa [8,9]. Despite the common molecular features described above, AMPs vary widely in many aspects, such as length, sequence and structure. As a result of such diversity, there is no universal target or mechanism of action for this class of compounds. Although specific mechanisms of action are a matter of debate, AMPs are often multifunctional. They may disrupt the membrane of pathogens, inhibit the activity of intracellular targets, and/or have immunomodulatory effects. This multitude of effects increases their efficacy and their capacity to avoid the development of resistance mechanisms [10,11].

Immunomodulation may in fact be the main action of antimicrobial peptides, leading some authors to re-name them as host defense peptides (HDPs) [12]. This designation fits with the fact that several AMPs have low activity in vitro but are effective against infections in vivo, sometimes even at lower peptide concentrations [11,12,13,14]. Moreover, at physiological salt concentrations, many AMPs lose their direct antimicrobial activity in vitro (due to charge neutralization), but keep the in vivo activity. Also, the minimal inhibitory concentrations calculated for killing in in vitro assays are very difficult to achieve in in vivo conditions. Therefore, AMPs probably act not exclusively by direct killing of the pathogen but also through the establishment of immune protective circuits. These immune protective circuits may include crosstalk between the innate and adaptive immunity. The reported immunomodulatory activities of HDPs include: modulation of the production of pro-inflammatory and anti-inflammatory cytokines and chemokines, recruitment of immune cells, induction of cellular differentiation and activation, regulation of cellular processes such as autophagy, apoptosis and pyroptosis, and also the promotion of wound healing [8,12,15]. In fact, reflecting the importance of their immunomodulatory activities, HDPs were shown to be involved in autoimmune disorders. Increased expression of cathelicidin and defensins is associated with different inflammatory conditions such as psoriasis, rosacea and others [12,16]. On the other hand, their unquestionable contribution to resistance to infection is evidenced by the correlation between decreased amounts of cathelicidin and increased susceptibility to infections in neutrophil-associated diseases such as morbus Kostmann [17].

In the context of infectious diseases, and particularly in intracellular infections, the combination of the diverse but complementary effects of AMPs is thought to be essential for efficient protective host response [9,16,18].

## 3. Defensins and Cathelicidins as Prototypic Host Defense Peptides

In mammals, two major classes of HDPs are known: the defensins (further classified as alpha- or beta-defensins, according to their patterns of disulphide bonds) and the cathelicidins. These peptides are abundantly produced by neutrophils, monocytes, mast cells, and epithelial cells, among others. In humans, alpha-defensins, namely human neutrophil peptides (HNP), comprise 30–50% of the total protein content in azurophil granules of neutrophils, which degranulate upon an inflammatory stimulus [8]. Beta-defensins can be expressed constitutively, as beta-defensin 1 (HBD1) in keratinocytes, or induced by inflammatory stimuli (e.g., Lipopolysaccharide (LPS) and Tumor Necrosis Factor Alpha (TNF-alpha)) as HBD2-4 in epithelial cells [8,19]. In the case of cathelicidins, humans and mice only have one cathelicidin gene, whereas others mammals, like cows, pigs, sheep and rabbits, can have several. These are produced as immature precursors that need to be proteolytically cleaved into mature active peptides, such as LL37 (in humans) or CRAMP (in mice, CRAMP—cathelicidin related antimicrobial peptide) [20,21]. Moreover, it has been shown that LL37 can be further cleaved into small active antimicrobial peptides [22,23]. LL37 is widely produced by epithelial and immune cells [24] and diverse microbial stimuli were shown to induce its upregulation [25,26,27,28,29]. This linear 37-amino acid alpha-helical peptide is one of the most extensively studied HDPs, with multiple antimicrobial and immunomodulatory activities. The importance of cathelicidin for host protection against infection is evident from the fact that mice lacking endogenous cathelicidin (CRAMP) are more susceptible to bacterial infections [28,30,31,32,33,34] whereas enhanced cathelicidin expression increased resistance [35,36,37]. Among the immunomodulatory activities of LL37, the impact on cytokine production is one of the best documented. Several studies revealed that LL37 impacts the macrophages’ capacity to produce pro-inflammatory cytokines, in response to Toll-Like Receptor (TLR) agonists. For example, LL37 decreases TNF production in response to LPS [38,39,40,41,42,43]. The mechanisms by which LL37 exerts this action are not clear and may be multi-factorial, including direct binding and neutralization of the TLR ligands and/or the activation of specific receptors (discussed below). This effect of LL37 on cytokine release may have both cell-intrinsic and systemic consequences for the control of intra-macrophagic infections. There are also several indications that HDPs, including LL37, have chemotactic activity, either directly or indirectly, through the induction of chemokine release [8].

Given the broad diversity of the immunomodulatory effects of HDPs, the existence of specific protein receptors has been postulated and investigated. As a result, several cellular receptors were suggested to be bound by LL37 and to mediate some of its effects (reviewed by [44]. *N*-formyl peptide receptor 2 (FPR2; formerly known as formyl peptide receptor like-1) was the first proposed functional receptor for LL37. FPR2 was characterized as the receptor mediating the chemotactic response to LL37 in human peripheral blood neutrophils, monocytes and T cells [45]. Additionally, LL37 was shown to increase neutrophil respiratory burst and extracellular trap (NET) responses through activation of FPR2 [46]. Another receptor thought to be activated by LL37 is P2X7, a membrane receptor expressed in several immune cells and involved in the inflammatory response, through the maturation and release of IL-1 and also through the induction of cell death. Elssner and colleagues demonstrated that LL37 acts as a P2X7 activator in LPS-primed monocytes [47]. Subsequent studies indicated that many of the immunostimulatory and immunomodulatory effects of LL37 could be explained by the specific activation of P2X7 [44]. Additionally, P2X7 is involved in the extracellular uptake of LL37 by human macrophages, where it promotes LTB4 and thromboxane A2 production or induces autophagy upon infection with intracellular pathogens such as *Mycobacterium tuberculosis* [48,49,50]. Some controversy remains as to the effects of cathelicidin in P2X7 activation, as it was shown that in mouse peritoneal macrophages CRAMP caused the inhibition and not the activation of P2X7 [44].

## 4. The Roles of Defensins and Cathelicidins in Host Defense against Mycobacteria

Mycobacteria are particularly challenging infectious agents. Their intrinsic impermeability, the intracellular localization, and slow proliferation rates, make them difficult to target with conventional antibiotics [51]. The extensive investigation of the role of AMPs in host defense against tuberculosis and other infections caused by mycobacteria has critically contributed to the understanding of the complexity of HDPs effects. Both defensins and cathelicidin were reported to inhibit the axenic growth of mycobacteria, although at relatively high concentrations [29,52,53,54]. Additionally, several studies revealed a strong upregulation of cathelicidin expression during mycobacterial infections both in vitro and in vivo [25,29,55]. More strikingly, cathelicidin was shown to be a crucial player in the historically recognized host protective effects of vitamin D against mycobacterial infection in humans [53,56]. Complementary work done by different research groups demonstrated that the activation of human macrophages by different agonists of the TLRs 2, 4 or 9 results in transcriptional induction of the vitamin D receptor (VDR) and vitamin-D-activating enzyme. Then, up-taken vitamin D is cleaved and activated, and will bind to VDR. The Vitamin D3–VDR complex induces cathelicidin expression and this induction is associated with an inhibition of the intracellular survival of mycobacteria [25,56,57,58,59]. In the absence of cathelicidin, vitamin D3 loses its inhibitory effect against mycobacteria [49,58]. Notably, vitamin D- induced cathelicidin production activates autophagy pathways in infected macrophages and this activation is necessary for the anti-mycobacterial effect [49,56,57,58,60], because autophagy antagonists block the anti-mycobacterial activities of LL37 [57,60].

Besides the induction of autophagy, another important effect of cathelicidin in mycobacteria-infected macrophages is the modulation of cytokine production. LL37 and analogues, decreased the production of pro-inflammatory cytokines such as IL-6, TNF-alpha, IFN-beta, IL-17 and IL-12p40, while increasing anti-inflammatory cytokines such as IL-10 and transforming growth factor-beta (TGF-beta), without affecting the antimicrobial capacity of the infected macrophages [54,60,61].

In the face of the clear anti-mycobacterial potential of cathelicidin observed in in vitro models, it is surprising that CRAMP knockout mice are not more susceptible to infection by mycobacteria than wild-type mice at early stages of infection [29,31]. In the case of *M. avium*, CRAMP knockout mice actually controlled better the infection, albeit transiently, than wild-type mice. This increased early resistance was probably related to an increased inflammatory response associated with infection [29]. In the case of *M. tuberculosis* infection, CRAMP-deficient mice revealed a decreased survival time and increased bacterial burdens in the lungs and spleen at later infection stages [31]. In this model, the absence of cathelicidin lead to defects on several cellular pathways such as calcium responsiveness, apoptosis and cytokine production, suggesting that increased susceptibility to infection was probably due to defects in immune signaling rather than to a reduced direct killing of the mycobacteria [31]. The lack of differences in mycobacterial loads in the organs of infected CRAMP knockout mice at early time-points may be related to the fact that virulent mycobacteria *per se* tend to downregulate the expression of cathelicidin in myeloid cells, as observed in some in vitro models [49,53]. Cathelicidin expression can however be increased through several stimuli. The most well described is vitamin D3, but other exogenous drugs can also upregulate LL37 production [25,29,55]. Importantly, cathelicidin regulation by vitamin D is a mechanism present in humans and other primates but not in mice, since the later lack the vitamin D response elements in the cathelicidin gene [62,63]. Thus care must be taken when addressing the roles of vitamin D, cathelicidin and autophagy in mice. Steiger and colleagues showed that glucocorticoids induced cathelicidin expression in human macrophages, independently of vitamin D, but they failed to promote autophagy, phagosome maturation and lysosome acidification. Curiously, in this case, even though cathelicidin was produced, no subsequent antimycobacterial activity was observed [64]. On the other hand, 4-phenylbutyrate induces LL37 and autophagy pathways, converging into the killing of intracellular mycobacteria [49].

Similarly to cathelicidins, defensins are also clearly involved in host protection against mycobacteria. Human alpha-defensin (or human neutrophil peptide, HNP) and beta-defensin 2 (HBD-2) were increased in the plasma and bronchoalveolar fluid (BALF) of patients with *M. avium-intracellulare* and *M. tuberculosis* infections [65,66]. Also, HBD-2 production was up-regulated in vitro by *M. tuberculosis* infection [67].

Bovine neutrophil beta-defensins 4 and 5 were shown to have antimicrobial activity against *M. smegmatis*, *M. bovis* and *M. tuberculosis,* both in axenic cultures and inside macrophages [68,69]. Human neutrophil peptide (HNP)-1 also decreased the growth of *M. tuberculosis* inside murine macrophages [70] and synergized with antibiotics, improving their microbicidal effect [71]. Interestingly, the culture of human monocyte-derived macrophages under hypoxic conditions resulted in increased levels of HBD-2 production and decreased mycobacterial growth [72]. Additionally, when the same type of cells were transfected with HBD-2, their antimycobacterial activity against *M. tuberculosis* increased [73].

Azurophil granule proteins from neutrophils extracts, which most probably include several HDP-like alpha-defensins, were able to improve the anti-mycobacterial activity of macrophages by increasing the co-localization of *M. bovis* -containing vacuoles with lysosomes, although no link to autophagy was observed [74]. The acquisition of granulocytic content by macrophages at inflammatory foci has been proposed as a cooperative mechanism by which both phagocytic cells contribute to host protection against mycobacteria [75].

## 5. Defensins and Cathelicidins in Other Intracellular Infections

Mycobacteria are not the only intra-macrophagic pathogens controlled by cathelicidins. The growth of *Salmonella enterica* serovar Typhimurium inside murine bone-marrow-derived macrophages was inhibited by CRAMP. This effect was due to an increased expression of CRAMP, dependent on the release of reactive oxygen species and host proteases upon infection. The mechanism by which CRAMP contributed to the inhibition of *Salmonella* proliferation was not elucidated, but impaired bacterial replication resulted in the intra-macrophagic formation of long filamentous bacteria [27]. Conversely, the treatment of macrophages with LL37 and LL37-derived nanoparticles increased their survival upon *Salmonella* infection [76]. Tang and colleagues showed that LL37, released from neutrophils, can be taken up by macrophages through the P2X7 receptor. Cathelicidin internalization resulted in increased reactive oxygen species and lysosome formation, culminating in the intracellular killing of *Staphylococcus aureus* [48]. The fate of phagocytized bacteria inside macrophages can be determined at early stages of internalization. In this context, it is of interest that LL37 enhances the phagocytosis of different pathogens. *Escherichia coli* and *S. aureus* were internalized more efficiently by THP-1 cells and human primary monocyte-derived macrophages treated with LL37, in a concentration-dependent manner. The combination of LL37 with HNP-1 resulted in an even more effective phagocytosis. This effect of LL37 was mediated through increased expression of FcγRs (CD68) and TLR4 [77] whereas HNP1-3 increased TNF-alpha and IFN-gamma, which then activated FcγRs (CD32 and CD64), leading to increased phagocytosis [78]. Fish cathelicidins also increase phagocytosis and respiratory burst of head kidney leukocytes. Moreover, these peptides are able to synergize with immunostimulant beta-glucans against *E. coli* infections [79]. Retrocyclins, humanized analogues of the teta-defensin peptides obtained from nonhuman primate leukocytes, were also able to bind to *Bacillus anthracis*, enhance phagocytosis and facilitate killing by RAW 264.7 macrophages [80].

In contrast, other peptides of the defensin family were found to decrease pathogen uptake. HD6 was shown to provide host defense against *Salmonella* challenges by forming peptide self-assembling aggregates described as “nanonets” that entrap bacteria in the intestinal lumen and thereby prevent bacterial invasion of the host epithelium and subsequent dissemination to other organ systems [81]. A similar mechanism may contribute to host defense against *Candida albicans*, *Listeria monocytogenes* and *Salmonella* mediated by HD6 [82].

A different mechanism underlies the effect of the alpha defensin HNP-1, which was found to decrease *Listeria monocytogenes* proliferation inside murine bone-marrow-derived macrophages by preventing the release and activity of listeriolysin O (LLO) [83]. In order to proliferate, *Listeria* relies on LLO, amongst other mechanisms, to disrupt the phagosomal membrane and escape to the macrophage cytosol where it will proliferate and invade neighbor cells. By impairing the activity of LLO, HNP-1 was able to prevent bacterial escape from the phagosomal harmful environment, cooperating with the macrophage antimicrobial mechanisms to control bacterial proliferation and dissemination [83].

## 6. Other Peptides, Other Bugs

Apart from defensins and cathelicidins, other HDPs were shown to modulate macrophage interaction with intracellular pathogens.

Lactoferricin peptides are known to have immunomodulatory properties, playing important roles both in the innate and adaptive immune responses. Lactoferricins induced apoptosis in several cancer cell lines without harming normal mammalian cells [84,85,86,87,88,89], and inhibited septic shock by binding to endotoxins [90]. Lactoferricin B, a lactoferrin-derived peptide, was shown to decrease macrophage uptake of *Listeria monocytogenes* and also to re-direct *Listeria*-infected macrophages from a necrosis to an apoptosis type of cell death [91,92]. A lactoferricin-derived peptide decreased intra-macrophagic survival of mycobacteria through the increase of phagosome–lysosome fusion and autophagy [93].

Another example of a macrophage-modulating peptide is the cecropin A-magainin hybrid peptide, which decreased the production of nitric oxide and TNF-alpha by RAW 264.7 cells in response to *E. coli* LPS [94]. Protegrin 1 and analogues also inhibited macrophages’ inflammatory response to endotoxins and polysaccharides from *Neisseria meningitidis,* by decreasing IL-1beta, TNF-alpha and nitric oxide [95]. Interestingly, protegrin 1 retained this effect in the absence of one of the two native disulphide bridges, which are mandatory for the antimicrobial activity of this peptide [95].

The liver-expressed antimicrobial peptide 2 (LEAP-2) decreases the *Edwardsiella tarda*-induced IL-1beta and TNF-alpha mRNA expression by fish kidney-derived monocytes/macrophages. Additionally, this peptide induces chemotaxis and an increased respiratory burst, critically contributing to the augmentation of the possibility of host survival post-infection [96].

## 7. Concluding Remarks

A large body of evidence has been gathered to prove that AMPs are crucial components of host defense against infection in diverse animal species. Better knowledge of the immunomodulatory effects of AMPs is very important not only to improve their antimicrobial potential but also to anticipate possible immune-related negative effects.

The importance of AMPs as potential alternatives to conventional antibiotics relies on the fact that these peptides represent a new antimicrobial paradigm. The antibiotics currently used in the clinic have a single defined target allowing for bacteria to develop resistance through discrete target alterations. At odds, antimicrobial peptides are characterized by the possibility of acting on multiple fronts by a variety of mechanisms, making the induction of resistance less likely. The current challenge is to translate this knowledge into the clinical inclusion of AMPs in therapeutic regimens. Important limitations have slowed this progress, including the high costs of peptide production, their low stability in vivo and undesirable side effects. Even so, important progress has been made in the last few years with different approaches to try to overcome these problems. The use of D enantiomers increases AMP resistance to proteases, and allows the sustaining of their activity for longer periods of time. The use of non-peptidic backbones (peptidomimetics), not only increases drug stability but also decreases the costs of production. Shorter sequences with improved activity also help to reduce the costs of production. Synergetic formulas of AMPs/HDPs with other drugs, or even with other HDPs, not only decreases the economic burden but also the possibility of undesirable side-effects and of resistance development. The conjugation with a specific antibody or receptor, and the encapsulation into micelles, liposomes or nanoparticles, allows the targeting of infected cells, improving the antimicrobial activity and decreasing side effects [12]. An increasing number of pre-clinical and clinical assays is underway to investigate the potential of AMPs to treat intracellular infections, but also to tackle other clinical problems such as wound healing/regeneration, and the induction or repression of HDPs to deal with auto-immune diseases, among others. For instance, brilacidin, a defensin-mimetic non-peptidic molecule, that shows antimicrobial activity against several different bacteria as well as immunomodulatory properties, is in late clinical development for use on oral mucositis, ulcerative proctitis/proctosigmoiditis and on acute bacterial skin and skin structure infections [97]. One interesting approach to consider is the stimulation of increased endogenous production of HDPs, either pharmacologically (for example with phenylbutyrate or vitamin D, as referred to previously) or through gene manipulation, as recently demonstrated in pigs [98]. Thus, considering the need for new antimicrobial drugs and the increasing evidence of the beneficial applications of HDPs, it will not take long before these molecules are more generally included in anti-infective therapeutic regimens.
